# Inhalations in Pectoral Diseases

**Published:** 1871-04

**Authors:** J. G. Westmoreland

**Affiliations:** Atlanta, Ga.; Professor of Materia Medica and Therapeutics in Atlanta Medical College


### INHALATIONS IN PECTORAL DISEASES.

BY J. G. WESTMORELAND, M. D., Professor of Materia Medica and Therapeutics in Atlanta Medical College.

Direct general or constitutional treatment for chronic diseases of the respiratory organs has long since been abandoned.

Incidentally, however, elective remedies become necessary in supporting that amount of general vigor essential to ultimate recovery. The usual amount of nourishment cannot, under such circumstances, be safely dispensed with; and to insure this, digestive stimulants and tonics may be sometimes required.

The nervous centers also require the aid of temporary and permanent excitants, in order to the proper amount of nervous energy. These are indirect restorative means. They keep the economy in a condition most favorable for the successful action of local remedies.

Various plans have been proposed for the introduction of agents, so as to come in contact with the respiratory tube at the diseased point. Each of these has its advocates. The article used, and the form of preparation, must, however, determine the mode in many instances. Previously to the late improvements in this practice, but after it had been ascertained that contact with the diseased surface was necessary to a cure, vapor inhalation was the usual plan. Of the remedies to be used, tinctures, decoctions, or infusions were made. The vapor rising from these preparations, when heated, being inhaled, was supposed to carry with it the medicinal agent which had been dissolved in the liquid, and thus to secure its contact with the diseased part. This is by no means certain, however. Substances not volatile, and which do not become so by heating, are left in the vessel after all the solvent has passed off in vapor. Only such articles as are volatile can be used successfully in this way, and then only when they are required in a diluted state.

Solids in the form of powder, when inhaled, do not generally reach points beyond the throat, owing to the abrupt change of direction required to enter the larynx. They are likely to come in contact with, and adhere to, the pharynx. All things considered, the form of fumes proves more convenient and useful than any other. The article in this way reaches the affected part in a sufficient degree of concentration to effect all the benefit of which it is capable. In this state, the remedy is readily carried, with the air, in respiration, into the minute bronchii.

Unfortunately, some of the most valuable catheretics are not subject to this form. Nitrate of silver, and other salts, applied locally for chronic inflammation, cannot be brought to this state; and while the combinations of chlorine with the metals and alkalies emit fumes when subjected to heat, yet they consist almost entirely of chlorine. Therefore, these combinations cannot be conveniently used by inhalation.

Iodine is in every respect admirably adapted to the local treatment of chronic diseases in the respiratory organs. As a catheretic, it is not inferior to most of the common applications for chronic inflammation and ulceration. In addition to this, as an elective agent it possesses catalytic properties, making it highly useful in the treatment of strumous and other constitutional affections, such as scrofula, tuberculosis and syphilis, by its solvent power upon the specific matters constituting these diseases. In advanced stages of the latter two affections, its introduction into the circulation for the "alterative" (catalytic) action is considered indispensable. Hence, iodine in some form is always given in secondary and tertiary syphilis. It is not unreasonable to suppose, therefore, that when directly applied to diseases resulting from the deposit of these specific substances, it may dissolve and lead to the catlaysis of any portions of them that may still remain at the point of irritation. This remedy is easily made to assume the form in which it readily reaches any portion of the respiratory tubes by inhalation, and without excessive dilution. By the application of heat, iodine, at once rising in fumes, passes out of the vessel containing it. No complicated or expensive apparatus is demanded for this purpose. A large mouthed vial, into which a grain or two of iodine is placed, may be held over a lamp or candle until the fumes rise so as to be inhaled. By a sudden forcible inspiration while the vial is held under one nostril, and the other closed with the finger, alternately, the whole respiratory mucous membrane may be impressed with the remedy. Indeed, when it is not desirable to affect the nares, this mode is perhaps preferable to that of inhaling through the mouth, on account of the readiness with which the fumes enter the larynx from the posterior nares. In view of all the facts above stated, it is reasonable to conclude that iodine is at least equal to any remedy, as a local application, in the treatment of chronic inflammation, irritation, or ulceration, in the respiratory apparatus.

It is not the object of this article to describe the modus operandi of catheretics in the cure of chronic structural lesions but to give reasons for selecting this particular article in the treatment of such local disease in the lungs and air passages. Iodine is not preferred so much for its superiority of action as for the facility with which it may be applied. On parts where liquids and solids can be safely and conveniently used, other articles may be found equally efficacious, and sometimes preferable.

The modes of introducing liquids into the wind pipe by injection and by saturated sponge with probang, are not only difficult to perform, but dangerous in results. These have given place to the more convenient and safe plan of atomization, by which liquids may be readily applied to the fauces and larynx. While to this mode of applying catheretic solutions for laryngeal and throat disturbance, there can be no well-founded objection; yet it should not perhaps be relied on when bronchial and pulmonic symptoms are to be met.

Chronic catarrh, in which sometimes the whole mucous surface of the air passages from the nares to the minute bronchia is the subject of irritation, requires the regular action daily of an efficient catheretic. In order to success, it is necessary that the remedy be applied throughout its entire extent. The fumes of iodine, taken in the manner above described, can be brought in contact with the whole surface without the introduction of an excessive amount.

Bronchitis, whether of tubercular origin or otherwise, requires also an alterative influence regularly applied, which may be afforded by the same means.

The same may be said of pulmonic lesions resulting from acute inflammation of the peranchyma, or from tuberculous deposit. Ulcerated portions of the lungs can in this way be so changed that the progress may be arrested.

In chronic catarrh or bronchorrhœa, dependent on an inactive state of the mucous membrane, the application of special excitants such as rosin may prove more effectual than catheretic means. This may also be used in the form of fumes by subjecting it to heat.

